# Exercise-Released Myokines in the Control of Energy Metabolism

**DOI:** 10.3389/fphys.2020.00091

**Published:** 2020-02-13

**Authors:** Claire Laurens, Audrey Bergouignan, Cedric Moro

**Affiliations:** ^1^CNRS, IPHC, UMR 7178, Université de Strasbourg, Strasbourg, France; ^2^Centre National d’Etudes Spatiales, Paris, France; ^3^Division of Endocrinology, Metabolism and Diabetes, Anschutz Health & Wellness Center, University of Colorado Anschutz Medical Campus, Aurora, CO, United States; ^4^INSERM, UMR 1048, Obesity Research Laboratory, Institute of Metabolic and Cardiovascular Diseases, Toulouse, France; ^5^Paul Sabatier University, University of Toulouse, Toulouse, France

**Keywords:** skeletal muscle, exercise, exerkine, crosstalk, metabolism

## Abstract

Physical activity reduces cardiometabolic risk, while physical inactivity increases chronic diseases risk. This led to the idea that exercise-induced muscle contraction contributes to metabolic regulation and health. It is now well established that skeletal muscle, through the release of endocrine factors, i.e., so-called myokines, crosstalk with metabolic organs such as adipose tissue, liver and pancreas. Recent advances suggested that a number of myokines are able to modulate adipose tissue metabolism and thermogenic activity, liver endogenous glucose production and β-cell insulin secretion. This novel paradigm offers a compelling hypothesis and molecular basis to explain the link between physical inactivity and chronic diseases. Herein, we review major findings and recent advances linking exercise, myokines secretion and inter-organ crosstalk. Identifying the molecular mediators linking physical activity to metabolic health could open the path toward novel therapeutic targets in metabolic diseases.

## Introduction

Physical activity reduces cardiometabolic risk, whereas sedentary behaviors favor the development of chronic diseases (Janiszewski and Ross, 2009; Booth et al., 2017; Powell et al., 2018). These observations led to the concept that exercise, by stimulating muscle contraction, participates in the regulation of energy homeostasis and organ function. It is now well known that skeletal muscle, through the secretion of endocrine factors, is able to communicate with several key metabolic organs involved in the control of energy metabolism (Pedersen and Febbraio, 2012). Recent advances emphasized the role of secreted proteins, i.e., myokines, which are able to regulate lipid mobilization from adipose tissue, liver endogenous glucose production, insulin secretion by beta pancreatic cells or to activate thermogenesis. In this review, we will describe the main metabolic effects of exercise-released myokines on main metabolic organs, i.e., skeletal muscle, adipose tissue, liver and pancreas. We will also discuss how this new paradigm offers a molecular basis to explain the link between sedentary behaviors and metabolic diseases.

## Skeletal Muscle Adaptations to Exercise

Physical inactivity is a major risk factor for obesity, type 2 diabetes, cardiovascular diseases, certain cancers, osteoporosis and early mortality (LaMonte et al., 2005; LaMonte and Blair, 2006). Aerobic capacity, or cardiorespiratory endurance, is a major predictive factor of cardiovascular mortality (Lee et al., 2010). Physical exercise is well-known to stimulate muscle oxidative capacity and lipid oxidation. Cross-sectional studies have demonstrated increased lipid oxidation in endurance-trained individuals compared to sedentary controls during an exercise bout with the same relative and absolute workload (Nordby et al., 2006). Longitudinal studies have shown that lipid oxidation is improved during an acute exercise bout performed after an endurance-training program for the same relative and absolute workload in either individuals with normal weight (de Glisezinski et al., 2003), overweight (Johnson et al., 2010) or obesity (Tarnopolsky et al., 2007). Even if several biochemical adaptations have been reported (Hawley et al., 2014), the greater mitochondrial oxidative capacity seems to be the main determinant. The increase in lipid oxidation during exercise is accompanied by an increase in the number of mitochondria and in mitochondrial enzymes activity in response to exercise training (Menshikova et al., 2005). Several studies have also demonstrated an increase in mitochondrial density through electron microscopy and in the expression of mitochondrial respiratory chain complexes (Menshikova et al., 2005; Engeli et al., 2012).

These molecular adaptations to training seems to be largely mediated by transcription factors such as peroxysome proliferator-activated receptor (PPAR)-gamma coactivator-1α (PGC-1α) and PPARβ, and metabolic sensors such as 5′-AMP activated kinase (AMPK). Thus, a skeletal muscle-specific overexpression of PGC-1α and PPARβ markedly improves oxidative capacity and endurance in mice (Lin et al., 2002; Wang et al., 2004). These animal models also display an improvement in insulin sensitivity and glucose tolerance. Several human studies also confirm the association between increased muscle oxidative capacity and insulin sensitivity in response to endurance or resistance training (Bruce et al., 2006; Toledo et al., 2007). However, most of the cellular and molecular mechanisms linking physical exercise to health benefits remain poorly understood. Identification of these mechanisms may lead to the discovery of new therapeutic targets in metabolic diseases but also allows the development of personalized exercise medicine (Neufer et al., 2015). Considering the benefits of physical exercise on many organs and in multiple chronic diseases, skeletal muscle has been longstanding considered as able to release humoral factors into the bloodstream. We will here describe the main myokines induced by physical exercise, i.e., exerkines, and involved in the regulation of energy metabolism.

## Myokines: Messenger of Skeletal Muscles to Remote Metabolic Organs

**FIGURE 1:**
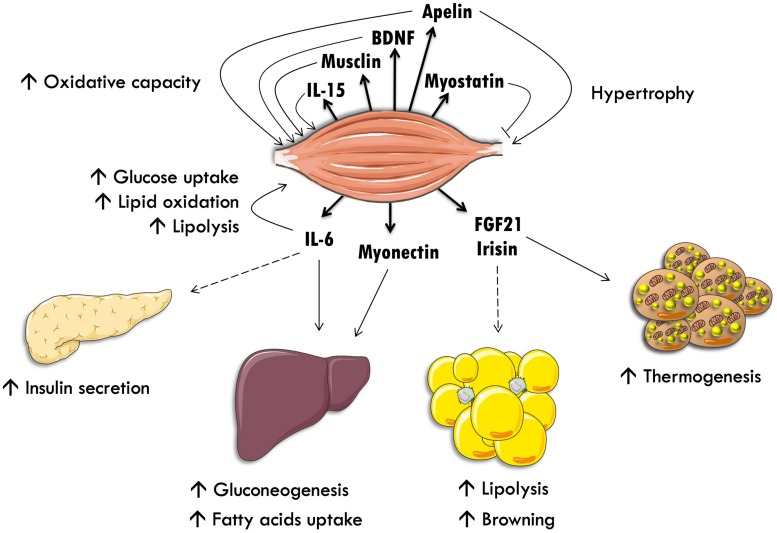
Skeletal muscle as an endocrine organ. Interleukin-15 (IL-15), brain-derived neurotrophic factor (BDNF) and interleukin-6 (IL-6) stimulate lipid oxidation and oxidative metabolism in an autocrine fashion. IL-6 also stimulates intramyocellular triacylglycerol lipolysis and glucose uptake. IL-6 stimulates pancreatic insulin secretion and exercise-induced hepatic neoglucogenesis through an endocrine communication. Myostatin inhibits muscle hypertrophy. Finally, fibroblast growth factor-21 (FGF-21) stimulates white adipose tissue lipolysis and brown adipose tissue thermogenesis.

By analogy with endocrine glands and adipose tissue, myokines are peptides, which are expressed, produced and secreted by muscle fibers. These myokines display autocrine/paracrine and endocrine effects, and could mediate the beneficial effects of exercise on other key organs involved in the regulation of energy homeostasis (Figure 1). The hypothesis that skeletal muscle produces secreted factors during exercise has been suggested when it was observed that muscle contraction induces physiological and metabolic adaptations in other organs, which are not mediated by the nervous system. Thus, an electric stimulation in skeletal muscle without nervous afference or efference in paraplegic patients recapitulates several of the physiological adaptations observed in valid individuals (Mohr et al., 1997).

By using different techniques, recent studies suggested that skeletal muscle may release several hundreds of proteins, although only a few have been characterized and their biological function demonstrated (Catoire et al., 2014; Hartwig et al., 2014; Deshmukh, 2016). Pourteymour et al. (2017) recently identified by RNA sequencing novel candidate myokines, and among those characterized colony-stimulating factor-1 (CSF1) as a novel exercise-regulated myokine. The biological role of CSF1 in the context of exercise still remains to be determined. Collectively, this indicates that skeletal muscle is therefore able to communicate with remote organs, through humoral factors secreted into the bloodstream during exercise.

## Myokines With an Autocine/Paracrine Action in Skeletal Muscle

Among these factors, we find the so-called myostatin, also known as *growth/differentiation factor 8*, whose gene invalidation leads to an excessive increase in muscle mass (McPherron et al., 1997). Myostatin seems to act localy by inhibiting muscle hypertrophy, but is also secreted in the bloodstream to act remotely on adipose tissue (McPherron and Lee, 2002). Other factors have been identified such as brain-derived neurotrophic factor (BDNF) (Rasmussen et al., 2009), musclin also called osteocrin (Subbotina et al., 2015), and interleukin-15 (IL-15) (Quinn et al., 2013) to control muscle hypertrophy and oxidative capacity in an autocrine fashion. At the muscle level, IL-6 seems to stimulate intramuscular triacylglycerols lipolysis and fatty acids oxidation in an autocrine manner (Wolsk et al., 2010). A human study has also demonstrated that IL-6 injection, at a dose reproducing physiological variations as to those observed during exercise, stimulates GLUT4 translocation to the plasma membrane and improves insulin sensitivity in skeletal muscle (Carey et al., 2006). This mechanism could involve the activation of AMPK. Many other proteins secreted by skeletal muscle with an autocrine effect have been identified, such as SPARC, fibroblast growth factor-21 (FGF-21), decorin, myonectin and irisin. Extensive reviews on this topic have been published elsewhere (Lee and Jun, 2019; Piccirillo, 2019).

More recently, we identified apelin as a novel myokine in human skeletal muscle. We showed that apelin gene expression is up-regulated in response to 8-week aerobic exercise training in obese male subjects (Besse-Patin et al., 2014). Apelin gene expression is up-regulated in human primary skeletal muscle cells in response to exercise-mimetic treatment including Ca^2+^ and cAMP releasing agents (Besse-Patin et al., 2014). It was further demonstrated that apelin is acutely released upon muscle contraction during exercise to act in an autocrine fashion to activate skeletal muscle hypertrophy. A progressive decline in muscle apelin expression and secretion with aging may contribute to the degenerative loss of muscle mass, quality and strength, i.e., sarcopenia (Vinel et al., 2018).

In a recent study, Furrer et al. (2017) demonstrated that B-type natriuretic peptide (BNP), which was previously thought to be mainly expressed in the heart (Moro and Lafontan, 2013), is also a PGC1α-dependent myokine released by contracting skeletal muscle to modulate macrophage function in a paracrine fashion. A tightly controlled crosstalk between muscle fibers and immune cells is thus a key event in training adaptation and for muscle regeneration and repair. This work resonates with the study of Subbotina et al. (2015) indicating that proteins belonging to the natriuretic peptide family may play an important physiological role in controlling muscle metabolism and function.

## Myokines With an Endocrine Action on Metabolic Organs

### White and Brown Adipose Tissues

Interleukin-6 was the first myokine to be discovered by Bente Pedersen and colleagues (Pedersen et al., 2003). Circulating IL-6 levels markedly increase in response to an acute exercise bout, up to 100-fold increase above resting level. This increase seems to be independent of muscle fiber damages and is tightly correlated with exercise intensity, duration and muscle mass involved in the mechanic workload (Suzuki et al., 2002). Several studies have demonstrated an increase in IL-6 expression and secretion in skeletal muscle in response to contraction, particularly when muscle glycogen content is depleted (Keller et al., 2001; Steensberg et al., 2001; Febbraio et al., 2003). This observation led to the suggestion that IL-6 is secreted by skeletal muscle in response to exercise and acts as a metabolic sensor. Thus, IL-6 could stimulate adipose tissue lipolysis and lipid mobilization during exercise to provide energy for muscle contraction and spare glucose (van Hall et al., 2003). An elegant clinical study has recently demonstrated that IL-6 is required to reduce visceral adipose tissue mass in response to exercise training in humans (Wedell-Neergaard et al., 2019). However, the role of IL-6 on adipose tissue lipolysis is still a matter of debate, as it was demonstrated that an acute elevation of IL-6 at a normophysiological level increased the rate of whole-body lipolysis due to an increase in muscle fatty acids release, whereas adipose tissue lipolysis remained unaffected (Wolsk et al., 2010). In addition, IL-6 does not display any acute effect on adipocyte lipolysis *in vitro*, as 2 days of treatment are required to induce glycerol release in culture adipocytes (Trujillo et al., 2004). Altogether, these data are therefore challenging the role of IL-6 in acute stimulation of adipose tissue lipolysis during exercise.

Fibroblast growth factor-21 is a new member of the fibroblast growth factor (FGF) family, which has been discovered in the early 2000s. Even if it has been primarily identified in the liver, FGF-21 is expressed in mouse and human skeletal muscle and seems to be regulated by insulin (Hojman et al., 2009). Circulating FGF-21 levels increases in response to a 2-week endurance training program (Cuevas-Ramos et al., 2012). FGF-21 displays anti-diabetic effects in mice through the stimulation of glucose transport in adipose tissue (Kharitonenkov et al., 2005) and stimulates lipolysis and thermogenesis in BAT (brown adipose tissue) (Inagaki et al., 2007; Hondares et al., 2010). FGF-21 has been described to regulate the expression of PGC-1α and favor the thermogenic program and browning in white adipose tissue (Fisher et al., 2012), as recently reviewed (Cuevas-Ramos et al., 2019). FGF-21 exhibits the characteristics of a metabolic myokine inducing an increase in energy expenditure and an improvement of insulin sensitivity in response to chronic exercise. It is important to note that some authors recently caught attention on potential detrimental effects of FGF-21 at high circulating plasma concentrations (Tezze et al., 2019). FGF-21 has been proposed as a biomarker of mitochondrial dysfunction in skeletal muscle and used as a predictor of disease progression. For instance, high circulating levels of FGF-21 are associated with high rates of mortality in end-stage chronic kidney diseases (Kohara et al., 2017).

Irisin is a myokine, which has been recently identified by the group of Bruce Spiegelman (Bostrom et al., 2012). As previously discussed, PGC-1α is a transcriptional co-activator induced by physical exercise, which activates mitochondrial biogenesis and muscle oxidative capacity. The authors hypothesized that PGC-1α stimulates the secretion of specific myokines, which are able to modulate the function of other organs. They identified irisin, which is the product of proteolytic cleavage of the membrane protein fibronectin type-III domain-containing protein 5 (FNDC5). FNDC5 is largely overexpressed in skeletal muscle of MCK-PGC-1α mice. Interestingly, browning of subcutaneous adipose tissue occurs in MCK-PGC-1α mice and a sharp increase in thermogenic genes such as uncoupling protein 1 (UCP1) and cell death-inducing DFFA-like effector A (CIDEA) is observed. The authors also showed that the treatment of differenciated primary adipocytes with recombinant irisin or with conditioned media from MCK-PGC-1α mice primary myocytes induces the expression of UCP1 and CIDEA *in vitro*. Irisin also stimulates the expression of PGC-1α and PPARα as well as basal and uncoupled respiration *in vitro* in murine subcutaneous adipocytes primary cell culture. The molecular mechanism by which irisin induces UCP1 could involve a partnership between PGC-1α and PPARα. Finally, a short 10-day treatment of wild-type mice with irisin induces an increase in UCP1 expression and the browning of subcutaneous adipose tissue. This treatment seems sufficient to increase total energy expenditure, slightly decrease fat mass and improve insulin sensitivity. These data seem to be physiologically relevant as irisin muscle expression and plasma level are increased in humans in response to a 10-weeks endurance training program or in older individuals (Timmons et al., 2012). However, the authors of this latter study did not observe significant variations in muscle irisin expression in younger subjects or in response to a resistance training program. Another longitudinal study also confirms the rise in circulating irisin level in response to an endurance training program in young healthy subjects (Huh et al., 2012). Altogether, irisin seems to be a metabolic myokine able to stimulate white adipose tissue thermogenesis, and thus contributes to energy balance control in response to physical activity in mice. This polypeptide may have a potential therapeutic interest in the management of obesity and its metabolic complications. However, the relevance of irisin in humans remains highly controversial, as some research groups have pointed out that the commercially available antibodies used to detect irisin might be unspecific (Albrecht et al., 2015). In addition, Jürgen Eckel’s group demonstrated that the translation of mice studies to humans might be compromised due to the presence of a stop codon in the gene encoding the precursor of irisin in humans (Raschke et al., 2013). Finally, other studies conducted in different groups of subjects (i.e., young versus old, lean versus overweight) and assessing different exercise modalities did not report a consistent rise of muscle irisin mRNA or circulating levels with exercise training, except for highly active elderly subjects (Timmons et al., 2012; Norheim et al., 2014).

A study from Lee et al. (2014) has shown that both irisin and FGF21 are able to increase mitochondrial respiration and induce thermogenesis *in vitro* when applied on human brown adipocytes from neck fat biopsies. The authors further demonstrated a fat depot-specific effect of FGF21 and irisin (or a combination of both). The beiging effect was greater in neck adipocytes compared to subcutaneous white adipocytes, and no effect was observed in visceral adipocytes. These data are intriguing when compared to the now well-accepted higher beiging ability of subcutaneous adipose tissue compared to visceral adipose tissue in humans (Zuriaga et al., 2017). Because irisin and FGF21 are released by muscle during exercise, one could hypothesize that exercise enhances WAT beiging. If such an effect has been observed in mice (Stanford et al., 2015), it is not the case in humans (Norheim et al., 2014). The effect of exerkines on thermogenesis in humans remains therefore an open question and further studies need to characterize the impact of exerkines on both WAT beiging and BAT activation in humans (Lehnig and Stanford, 2018).

Myonectin, also known as C1q tumor necrosis factor α-related protein isoform 15 (CTRP15), was identified as a nutrient-sensitive myokine by Seldin et al. (2012). It has been demonstrated that myonectin is an exercise training-responsive myokine. Myonectin plasma level is increased in response to an 8-week aerobic exercise-training program in humans (Pourranjbar et al., 2018) and muscle myonectin gene and protein expression are both induced after treadmill endurance exercise in mice (Rabinovich-Nikitin and Kirshenbaum, 2018). Myonectin has been shown to target adipose tissue, but its metabolic role in response to muscle contraction has not been clearly established yet. Myonectin deletion impairs lipid handling resulting in an increased adipose tissue mass in diet-induced obese mice. However, these changes are independent of modifications in adipose tissue lipolysis in myonectin knockout mice, and are due to increased lipid uptake into adipocytes in postprandial conditions (Little et al., 2019). Furthermore, myonectin does not seem to be required for the physiological responses to exercise in mice (Little et al., 2019). Therefore, its role in the context of exercise warrants further investigations.

Collectively, adipose tissues appears to be important targets of myokines controlling energy fluxes and fuel availability to the contracting muscle. Some of these myokines can also improve adipocyte oxidative capacity and glucose uptake, a physiological adaptation contributing to endurance exercise-mediated metabolic health improvement. These myokines also seem to activate thermogenesis through a direct action on BAT. However, caution should be taken when interpretating the relevance of thermogenesis in mice, since some studies are not performed at thermoneutrality but instead at room temperature (i.e., almost 21°C). The thermal stress induced by temperature below thermoneutrality increases metabolic rate beyond what is observed in humans in the resting state. Therefore caution should be taken when translating phenotypes in mice to humans.

### Pancreas

We recently observed a significant increase in glucose-stimulated insulin secretion from isolated mice islets in response to conditioned media from resting non-treated human primary myotubes. This indicates that skeletal muscle cells produces basal rates of secreted factors capable of modulating insulin secretion (Mizgier et al., 2017).

Previous studies suggested a potential crosstalk between skeletal muscle and pancreatic β-cell through IL-6 (Handschin et al., 2007; Ellingsgaard et al., 2011). In the first study, authors observed an elevated IL-6 secretion rate from muscle-specific PGC-1α knockout mice. They further showed that this translated into higher plasma IL-6 levels disrupting insulin secretion by pancreatic β-cell. In contrast, the study by Ellingsgaard et al. (2011) suggested that IL-6 released by contracting muscle can induce glucagon-like peptide 1 secretion by intestinal L cells and pancreatic α-cells, which consequently promotes insulin secretion. The discrepancy between the two studies may lie in the various physiological contexts behind the studied mouse models. In a context of systemic inflammation and lipotoxicity, chronic elevation of IL-6 could have detrimental effects on insulin secretion by altering the positive regulation of β-cell by other hormones. Physiologically, insulin secretion is expected to decrease during acute exercise to relieve a break on lipolysis and hepatic glucose production. Controversies regarding the role of IL-6 as a myokine on insulin secretion therefore still exist and further research is needed.

In a recent study, it was shown that the PGC1α-dependent myokine irisin (FNDC5) behaves as a novel pancreatic β-cell secretagogue (Natalicchio et al., 2017). Interestingly, irisin enhanced insulin biosynthesis and glucose-stimulated insulin secretion in human and rat pancreatic β-cells. More interestingly, human skeletal muscle cells cultured under lipotoxic conditions increased irisin secretion. These conditioned media containing irisin were able to reduce β-cell apoptosis under lipotoxic stress induced by palmitate treatment. Altogether, these data show that irisin promotes β-cell survival and contributes to a crosstalk between skeletal muscle and β-cell under lipotoxic stress.

In conclusion, these studies highlight the existence of a crosstalk between skeletal muscle and pancreatic β-cell, which may be relevant in physiological conditions and diseases state.

### Liver

Pedersen and Febbraio nicely showed that recombinant human IL6 infusion in lean healthy subjects during moderate intensity exercise increases hepatic glucose production to the same extent as those observed during high intensity vigourous exercise (Febbraio et al., 2004). This demonstrates that IL-6 has the ability to enhance hepatic glucose production when released by contracting skeletal muscle during exercise. Seldin et al. (2012) showed that myonectin improves systemic lipid metabolism by increasing liver fatty acid uptake through up-regulation of fatty acid transporter genes such as cluster of differentiation 36 (CD36), fatty acid transport protein (FATP) and fatty acid binding protein (FABP) (Seldin et al., 2012). In summary, only few exerkines have been shown to modulate liver functions. Future studies will likely identify novel exercise-regulated myokines capable to crosstalk with the liver.

### Brain

The brain plays a central role in the integration of whole-body afferent signals (nervous, hormonal, and nutritional), including muscle signals in the context of exercise, and sends downstream efferent messages to peripherical metabolic organs. A number of myokines including cathepsin B, irisin, BDNF and FGF-21 have been suggested to play a role in the muscle-brain crosstalk (Delezie and Handschin, 2018). They do not seem to induce efferent signals to peripherical metabolic organs but rather to mediate exercise-induced improvement of brain functions such as cognition, memory, neuroplasticity, motor coordination, sleep, and mood. Some of these effects are mediated through neuroprotection and reduction in brain inflammation. This goes beyond the scope of this review and has been detailed elsewhere (Moon et al., 2016; Delezie and Handschin, 2018; Kim et al., 2019). FGF-21 seems to be able to cross the blood-brain barrier and to modulate sympathetic nerve output to brown adipose tissue in rodents (Owen et al., 2014). Further studies are required to better understand the muscle-brain crosstalk and brain-mediated effects of these myokines on remote metabolic organs, particularly in humans.

## Impaired Myokine Secretion as a Trigger of Adverse Health Effects Linked to Sedentary Behaviors?

It is well established that physical inactivity (<150 min/week of moderate-to-vigorous physical activity) and sedentary behaviors like sitting increase the risk of type 2 diabetes, a number of cancers, cardiovascular diseases and other chronic diseases. Because myokines allow muscle contractions to modulate other tissues through a pathway independent of the nervous system (Kjaer et al., 1996), the adverse health effects of physical inactivity and sedentary behaviors may result from the lack of muscle contraction. Given the anti-inflammatory effects of the myokines secreted in response to exercise, physical inactivity may lead to inflammation and inability to store fat in subcutaneous adipose tissue. Muscle unloading during bed-rest studies has been associated in healthy adults with a shift toward pro-inflammatory conditions (Rudwill et al., 2013; Mutin-Carnino et al., 2014) and an increase in visceral adipose tissue (Belavy et al., 2014). While concomitant exercise can prevent the increase in pro-inflammatory markers (Rudwill et al., 2013; Mutin-Carnino et al., 2014), no effect of resistive exercise was reported on body fat redistribution (Belavy et al., 2014). Similarly, resistive exercise training did not prevent the development of insulin resistance, hypertriglyceridemia and alterations in nutrient oxidation by bed-rest-induced physical inactivity (Bergouignan et al., 2006). These metabolic alterations were, however, mitigated when aerobic exercise was performed in addition to resistive exercise suggesting either the existence of a dose-response relationship between the number of muscle contractions and the health benefits, or that other factors such as activity or total energy expenditure have effects on metabolism in addition to or in synergy with muscle contractions. Further studies are warranted to better understand the respective contribution between lack of muscle contraction and low energy expenditure in the development of metabolic diseases associated with physical inactivity.

## Where Do We Go From Here?

One of the major challenges in the next few years will be to address whether the findings observed in animal and cell models can be translated to human physiology. In addition, many novel myokines still remain to be identified and the currently described set of myokines probably only reflects the tip of the iceberg. Given the beneficial effects of exercise on adipose tissue remodeling, we believe research efforts should be placed on the identification of novel exercise-induced myokines that may control adipose tissue metabolism and function. As physical activity is known to influence appetite and energy intake (Beaulieu et al., 2016; Thivel et al., 2016), understanding the role of the muscle secretome on the central control of appetite and energy intake is another important field of research to pursue. Finally, it is still unclear whether all myokines are required to confer the beneficial adaptations to exercise. Future studies should better characterize the respective role of each myokine and assess their potential additive or synergistic contribution to whole-body phenotype.

## Conclusion

Skeletal muscle behaves as an endocrine organ through the secretion of myokines and modulates the function of key metabolic organs in response to exercise. This original crosstalk between skeletal muscle and metabolic organs provides a conceptual basis to understand how exercise lowers the risk to develop several chronic diseases and increases health- and life-span. Highlighting the potential therapeutic role of novel metabolic myokines emphasizes the importance of inter-organ crosstalk mediated by exercise. It now seems well established that some exerkines mediate the beneficial physiological adaptations of exercise in the control of energy homeostasis and insulin sensitivity. It is therefore reasonable to think that physical inactivity and conditions altering muscle integrity (neuromuscular diseases, aging…) impair the secretion of various metabolic myokines, favoring in the long term the development of chronic diseases such as obesity, diabetes and cardiovascular diseases.

## Author Contributions

All authors listed have made a substantial, direct and intellectual contribution to the work, and approved it for publication.

## Conflict of Interest

The authors declare that the research was conducted in the absence of any commercial or financial relationships that could be construed as a potential conflict of interest.
